# Can the Blood Alcohol Concentration Be a Predictor for Increased Hospital Complications in Trauma Patients Involved in Motor Vehicle Crashes?

**DOI:** 10.3390/ijerph7031174

**Published:** 2010-03-18

**Authors:** Jaime H Kapur, Victoria Rajamanickam, Michael F Fleming

**Affiliations:** 1 University of Wisconsin Department of Surgery, Clinical Science Center H4/7, 600 Highland Ave, Madison, WI 53792, USA; 2 University of Wisconsin Department of Surgery Biostatistiscs, 5105 Wisconsin Institutes for Medical Research (WIMR) Building 1485, 1111 Highland Avenue, Madison, WI 53792, USA; E-Mail: rajaman@surgery.wisc.edu; 3 University of Wisconsin Department of Family Medicine, UW Department of Family Medicine 1100 Delaplaine Ct., Madison, WI 53715, USA; E-Mail: mffemin@wisc.edu

**Keywords:** alcohol intoxication, alcohol withdrawal delirium, trauma, motor vehicle, complications

## Abstract

The goal of this report is to assess the relationship of varying levels of blood alcohol concentration (BAC) and hospital complications in patients admitted after motor vehicle crashes. Data for the study was collected by a retrospective review of the University of Wisconsin Hospital trauma registry between 1999 and 2007 using the National Trauma Registry of the American College of Surgeons (NTRACS). Of 3729 patients, 2210 (59%) had a negative BAC, 338 (9%) <100 mg/dL, 538 (14%) 100–199 mg/dL, and 643 (17%) >200 mg/dL. Forty-six percent of patients had one or more hospital related complications. The odds ratio (OR) for the occurrence of alcohol withdrawal in the three alcohol groups compared to the no alcohol group was 12.02 (CI 7.0–20.7), 16.81 (CI 10.4–27.2), and 30.96 (CI 19.5–49.2) as BAC increased with a clear dose response effect. While there were no significant differences in the frequency of the total hospital events following trauma across the four groups, rates of infections, coagulopathies, central nervous system events and renal complications were lower in the high BAC group. Prospective studies are needed to more precisely estimate the frequency of hospital complications in patients with alcohol use disorders and in persons intoxicated at the time of the motor vehicle accident. The study supports the use of routine BAC to predict patients at high risk for alcohol withdrawal and the early initiation of alcohol detoxification.

## 1. Introduction

Over 3.6 million people are injured annually in crashes involving a motorized vehicle [1] and they represent the majority of trauma patients arriving at Level 1 trauma centers in the U.S. [2,3]. Alcohol is reported to be involved in 19%−38% [2,4–8] of crashes with 25−30% of victims likely to be alcoholics [9–11]. Given the high prevalence of alcohol use in these patients, using the blood alcohol concentration (BAC) in conjunction with other screening tests can identify patients who may benefit from brief alcohol intervention, resulting in decreased motor vehicle events and health cost savings [12,13]. However, utilizing the BAC as a clinical predictor for patient outcomes is less clear, particularly in relation to the level of BAC on admission.

There are conflicting findings regarding the influence of alcohol on injury severity, mortality, and morbidity indicators such as length of hospital stay, need for ICU care or ventilatory support, requirement of blood products or surgery, and in-hospital complications [14]. Some reports show a positive BAC predicts increased injury severity, mortality [15,16] and overall morbidity [17]. Other studies refute an independent association between alcohol and higher morbidity [18–21], instead citing greater injury severity as the culprit [22]. Some only find higher morbidity in chronic alcohol abusers [23] or patients with alcohol withdrawal syndrome [24].

These studies have compared only two groups of patients, those with a positive BAC (often defined as >100 mg/dL but sometimes with a lower cutoff) and those with a negative BAC. Only two studies have used graded intoxication cutoff levels (e.g., <100 mg/dL, 100−200 mg/dL, *etc*.) to evaluate mortality or the occurrence of complications [23,25]. A few other independent studies have documented a nonlinear relationship between levels of intoxication and their potentiating effect on injury severity [16,26]. Therefore, given the disparate findings about trauma patient outcomes when considering the patient’s intoxication level, this study aims to evaluate the differences in complications rates and short term outcomes in motor vehicle crash victims relative to their BAC.

## 2. Methods

Patients included in this study were from a single, academic, tertiary Level I trauma center. Data for the study was collected by a retrospective review of our National Trauma Registry of the American College of Surgeons (NTRACS) from July 1, 1999 to December 31, 2007. All patients 18 years of age or older who were admitted to our trauma center after a motorized vehicle collision and had their blood alcohol concentration (BAC) determined on arrival in the emergency department were included. This cohort was divided into four groups based on their BAC result. These groups included patients with a negative BAC, a BAC less than 100 mg/dL, a BAC of 100–199 mg/dL, and a BAC of greater than 200 mg/dL.

There were 5592 trauma patients involved in a motor vehicle collision (either car, truck, or motorcycle) over the study period. Obtaining a BAC on all trauma patients is now part of our trauma protocol, however testing was at the discretion of the trauma attending prior to 2003 and was not routinely done during the earlier years of the study period. Hence, not all of these patients had a BAC documented and only those with one were included for analysis (n = 3729). The trauma patients that did not have a BAC and who were not included in our analysis were more likely to be female (45% *vs.* 31%), older (43 *vs.* 39), have a lower injury severity score (14.1 *vs.* 16.9), fewer hospital days (7.7 *vs.* 8.8) and more likely to arrive from another hospital (48.6% *vs.* 39.1%).

Variables collected from the database included age, sex, ISS (injury severity score), Glasgow Coma Score (GCS) on arrival, length of hospital stay, length of intensive care unit (ICU) stay, complications in care, discharge disposition, and mortality. Additionally, whether the patient arrived from the scene or was transferred from a referring hospital, what type of vehicle the patient was in at the time of the collision (4 wheeled *vs.* motorcycle), the patient’s position in the vehicle (driver *vs.* passenger), and whether the patient was using a safety device (helmeted, restrained, none, or other) was also queried. Complications were defined using specific NTRACS criteria and extracted from patient charts by trained trauma database personnel. The complications evaluated were grouped in the following broader categories: alcohol withdrawal, central nervous system (CNS), cardiac, pulmonary, coagulopathy, gastrointestinal (GI), infectious, and renal (Table 1).

The comparison of suspected risk factors between alcohol levels, as well as the association between suspected risk factors and complications was assessed using Chi-square test for discrete variables. When the variables of interest were continuous a non parametric version of T test, Wilcoxon rank sum test was used in order to better meet the assumption of normality. Further, multivariate logistic regression analyses were performed to investigate the relationship between all three intoxication states and occurrence of complications after adjusting for other confounding factors. The negative BAC group was used as the reference group. The difference in injury severity scores between BAC groups was compared using an analysis of variance (ANOVA) and pair-wise comparisons were based on Fisher’s protected least significance difference tests. All data were rank-transformed prior to analysis in order to better meet the assumptions of ANOVA. All P values reported were 2 sided; P-value less than 0.05 was considered significant. A Bonferroni adjustment was performed when comparing intoxication status with the reference group so that P-values of less than 0.005 were considered significant. Analyses were performed using SAS statistical software version 9.1 (SAS institute Inc. Cary, NC).

## 3. Results

Complete patient characteristics are presented in Table 2. The majority of patients were men with the proportion of men in each group increasing as BAC increased. The mean age for those with negative BAC’s was 42.0, while the mean ages for the alcohol positive groups were younger (33.6, 32.4, and 34.9 respectively). About 80% of the collisions seen in all groups involved a car or truck with patients from the highest BAC group more likely to be in a car or truck than patients from all other groups. The majority of patients in all groups were drivers rather than passengers. Patients from the highest BAC group were more likely to come to our trauma center from the scene, rather than be transferred from a referring hospital. Mortality across all groups was 4–5%. Sixty-six to 76% of patients were discharged to home, which may have included home health nursing; up to 25% went to a rehabilitation or skilled nursing facility; and 3–6% went to jail, left against medical advice, or requested transfer to a different facility.

Of the 3729 patients, 1733 (46%) had some type of complication during their hospital course with an increasing incidence seen as the BAC increased (Table 3) with 44% no alcohol group reporting a complication and 51% of the BAC > 200 reporting a complication. While there were no significant differences across the four groups in frequency of “any complications”, there were higher rates of alcohol withdrawal in the positive BAC groups and fewer infectious disease, renal, CNS, and coagulopathy complications in the high alcohol group compared to the negative BAC group.

**Figure 1.**
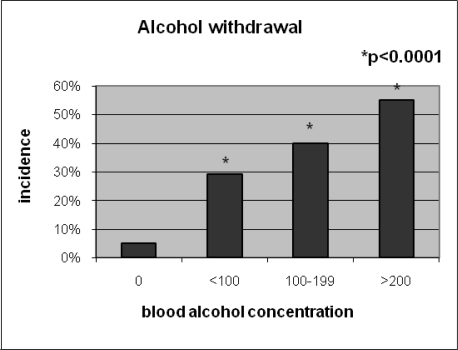
Incidence of alcohol withdrawal syndrome as BAC increases.

The occurrence of alcohol withdrawal correlated with the BAC as there was a 5% incidence in the no alcohol group that increased linearly to 55% in those with the highest BAC’s (Figure 1).

After adjusting for age, sex, the patient’s position in the vehicle, and whether the patient was transferred or from the scene, the odds ratio (OR) for the occurrence of alcohol withdrawal in the alcohol groups compared to the no alcohol group was 12.02, 16.81, and 30.96 (Table 4). The multivariate analysis found the opposite relationship with four other hospital complications with the higher BAC group. This group had significantly lower frequency of infectious complications, coagulopathy, renal complications, or a CNS complication. No significant differences were observed among the groups for pulmonary, cardiac, and GI complications.

## 4. Discussion

This study supports the determination of the BAC as part of routine care in patients admitted for motor vehicle collision trauma. We found a strong dose response effect between BAC and risk of alcohol withdrawal. Patients with a BAC > 200 mg/dL had 30 fold risk (OR = 30.96, 19.5–49.2) of withdrawal and those with a BAC < 100 mg/dL had a 12 fold risk (OR = 12.02, 7.0–20.7) compared to persons with a negative BAC. While BAC has been shown to be a significant predictor for alcohol withdrawal [27], this is the first study to demonstrate the dose response effect between high BAC’s and the increasing probability of withdrawal. Our incidence of alcohol withdrawal across all groups was slightly lower than reports of 59–67% in trauma patients from other studies [28,29], however these studies only looked at ICU patients while we included all trauma admissions with most patients admitted to general surgical units.

The second primary goal of the study was to determine if there was a dose response effect between increasing BAC and other hospital complications commonly seen in patients admitted for trauma. We hypothesized that patients with higher BAC’s would be at greater risk for complications such as bleeding, infection, respiratory problems, and thromboembolic events. Laboratory studies have reported that alcohol causes increased susceptibility to pulmonary and gastrointestinal infections [30], impairs immune defense [31], and has other deleterious physiologic effects [32]. While our data did not demonstrate an increased overall risk of hospital related complications [19,21,23] there were differences in the frequency of four events in favor of the high BAC group.

As reported in table 3 and 4 there was a statistically significant decrease in the frequency of infections, CNS complications, renal changes and coagulapathy in the high BAC compared to the negative BAC group. There a number of potential explanations for the finding including selection bias, measurement issues inherent in retrospective trauma data bases, co-morbid chronic illnesses, better medical care in the high BAC group, or a physiological protective effect. Regarding selection bias we compared trauma patients in the data base who had a BAC with those that did not and found that the no BAC group were younger, more likely to be female and had less severe injuries. This finding shows that the two groups were different and that there may be some selection bias, however the bias does not explain lower rates of some complications in the high BAC group. Additionally, the possibility of a type I error was addressed with a Bonferroni adjustment and we still found a statistically significant decrease in the risk of complication with a high BAC when compared to a negative BAC. However, this data does not address the issue that a disproportionate number of trauma victims with high BAC may have died at the scene of the accident. The only way to minimize the selection, measurement and confounding biases inherent in a trauma data base study, such as the one conducted, is to conduct a prospective study. The question remains open as to the dose response effect of a high BAC on hospital complications.

What are the clinical implications of the study? First the study supports the use of routine blood alcohol concentration determination on all patients admitted for motor vehicle related trauma. This information allows inpatient services to start benzodiazepines early before the development of severe withdrawal. Studies have shown that trauma patients in withdrawal are much more likely to go undiagnosed compared to medical or psychiatric inpatients [33,34]. Additionally, patients with alcohol withdrawal syndrome have been shown to incur longer hospital and ICU stays, more complications, and greater hospital costs [24,28,35].

Second, patients with a positive blood alcohol concentration may not be at greater risk for trauma related complications. Most studies have shown they are just as severely injured [17–20,23,25] or possibly more injured [16,22] than the non-alcohol group. Based on our findings, careful monitoring of these patients is indicated more for the high risk of alcohol withdrawal syndrome than for any greater risk of other complications. Some have also shown that intoxicated patients may be more likely to have some of their injuries initially undiagnosed which would support the use of BAC’s to identify these patients as well [22].

Finally, the finding that non-intoxicated patients were actually at a higher risk for infections, coagulopathies, renal impairment and CNS complications may point to other natural disease processes that contributed to their hospital course or even the cause of their MVC. These processes may include things like diabetes, sleep-apnea related drowsiness, heart disease, seizures, dizziness, and the effects of certain medications. While a positive BAC is certainly a red flag for complications related to alcohol withdrawal, the lack of a positive BAC should alert physicians to be wary of other medical conditions that can lead to complications.

Strengths of the study include a large sample, data obtained from a well established longitudinal trauma registry, a study conducted in a level 1 trauma center located in one of the highest drinking states in the country, and careful analysis. There are a number of limitations of the study including the use of retrospective data, the location of the hospital in a low density population center, the change in policy during the selected study period from discretionary BAC’s to routine BAC’s for all trauma patients, missing information in the trauma database, and limited information on alcohol use disorders and prior health status of the subjects.

As discussed previously, the effects of using retrospective data are selection, measurement, and confounding biases. Being in a low density population center limits the applicability of our findings when considering centers serving larger or smaller populations than ours. Additionally, our average population is mostly Caucasian with African-American, Latino, and other minorities less represented than in more urban Level 1 trauma centers. It is unfortunate that BAC’s were not routinely performed during the earlier years of the selected study period. This limitation could be eliminated by choosing a different study period or setting up a prospective collection, however we showed that while the no BAC group was different, these differences did not affect the outcomes we analyzed. There was also missing data on hospital complications from a number of subjects since the data was not collected to answer our primary hypotheses. A prospective study specifically designed to answer these hypotheses would be less likely to miss data points, be more tailored to collect details of interest, and be less likely to misclassify data. For example, while the UW hospital has had a standard alcohol withdrawal protocol in place prior to the trauma data base, a diagnosis of alcohol withdrawal was sometimes difficult to determine. The definition of “alcohol withdrawal” was broad when the initial data was extracted from the medical record, leading to possible misclassifications during subsequent analysis.

## 5. Conclusions

Since alcohol use is a common and persistent problem in patients involved in motor vehicle collisions, our study demonstrates the importance of routinely obtaining a BAC on these patients. As the BAC rises, these patients are at greater risk for alcohol withdrawal syndrome and clinicians should have a low threshold to start prophylactic medications. Overall, intoxicated patients do not seem to be at a greater risk for other hospital complications; however, others have shown that those with alcohol withdrawal syndrome do not follow this pattern. Prospective studies are needed to more precisely estimate the frequency of hospital complications in patients with alcohol use disorders and in persons who were acutely intoxicated at the time of the motor vehicle accident.

## Figures and Tables

**Table 1.** List of specific complications associated with each general complication group.

**Complication group**	**Specific complications**
Alcohol withdrawal	If patient required treatment for signs or symptoms of withdrawal or had an alcohol withdrawal treatment sheet in their chart
CNS	Brain death, anoxic encephalopathy, seizure, CVA
Cardiac	Arrhythmia, CHF, pulmonary edema, cardiac arrest, cardiogenic shock, MI, pericardial effusion, pericardial tamponade
Pulmonary	ARDS, aspiration, pleural effusion, respiratory failure, pneumothorax, atelectasis
Coagulopathy	Coagulopathy, pulmonary embolism, DIC, DVT, other thrombosis
GI	Upper or lower GI hemorrhage, peptic ulcer, ileus, peritonitis, obstruction
Infection	Septicemia or sepsis-like syndrome, pneumonia, urinary tract infection, other infection (abscess, cellulitis, empyema, gangrene, graft infection, intra-abdominal abscess, line infection, yeast infection, wound infection, meningitis, orthopedic wound infection, osteomyelitis, other infection, ventriculitis)
Renal	Renal failure, miscellaneous renal complications

CNS = central nervous system; CVA = cerebrovascular accident; CHF = congestive heart failure; DIC = disseminated intravascular coagulation; DVT = deep vein thrombosis; GI = gastrointestinal; MI = myocardial infarction.

**Table 2.** Patient characteristics (n = 3729).

	**No ETOH** (n = 2210)	**BAC < 100** (n = 338)	**BAC 100–199** (n = 538)	**BAC ≥ 200** (n = 643)
**Male**	1179 (61%)	234 (78%)	349 (78%)	445 (82%)
Age *	42.0 (18.5)	33.6 (13.4)	32.4 (11.5)	34.9 (11.5)
GCS *	13.1 (4.9)	12.4 (5.1)	12.3 (5.2)	12.4 (5.0)
ISS *	17.3 (13.2)	16.5 (12.6)	14.0 (13.5)	14.4 (13.5)
Hospital days *	9.4 (13.1)	8.6 (12.2)	8.8 (13.5)	6.7 (10.9)
ICU days *	2.4 (5.9)	2.3 (5.2)	2.7 (6.5)	1.9 (4.9)
**Discharge disposition**				
Died/Hospice	119 (5.4%)	18 (5%)	22 (4%)	27 (4%)
Home	1468 (66%)	239 (71%)	379 (70%)	490 (76%)
Rehab or Nursing facility	543 (25%)	70 (21%)	106 (20%)	91 (14%)
Jail/AMA/other transfer	80 (4%)	11 (3%)	31 (6%)	35 (5%)
**Type of vehicle**				
Car/Truck	1777 (80%)	265 (78%)	447 (83%)	572 (89%)
Motorcycle	433 (20%)	73 (22%)	91 (17%)	71 (11%)
**Position in vehicle**				
Driver	1497 (78%)	210 (70%)	348 (76%)	458 (85%)
Passenger	388 (21%)	86 (29%)	87 (19%)	66 (12%)
Unknown	39 (2%)	4 (1%)	14 (3%)	16 (3%)
**Patient arrived from:**				
Scene	1296 (59%)	117 (49%)	215 (52%)	443 (81%)
Referring Hospital	909 (41%)	123 (51%)	198 (48%)	102 (19%)
Clinic/Home	5 (0.2%)	0 (0%)	1 (0.2%)	2 (0.3%)

* mean ± standard deviation.

ETOH = alcohol, BAC = blood alcohol concentration, GCS = Glasgow Coma Score, ISS = Injury Severity Score, ICU = Intensive Care Unit, AMA = Against Medical Advice, N/A = not available.

**Table 3.** Incidence of patient complications among all groups.

	**No ETOH** (n = 2210)	**BAC < 100** (n = 338)	**BAC 100–199** (n = 538)	**BAC ≥ 200** (n = 643)	**Total**
**Any complication *(n*)**	44% (980)	49% (167)	48% (260)	51% (326)	**46% (1733)**
**Alcohol WD**	5% (46)	29% (49)	40% (104)	55% (181)	**22% (380)**
**Pulmonary**	24% (238)	21% (35)	22% (57)	19% (62)	**23% (392)**
**CNS**	11% (111)	6% (10)	5% (13)	6% (20)	**9% (154)**
Cardiac **	18% (178)	11% (18)	9% (22)	9% (28)	**14% (246)**
Coagulopathy **	18% (173)	17% (29)	15% (38)	7% (23)	**15% (263)**
**GI**	9% (97)	9% (16)	8% (20)	6% (18)	**9% (151)**
Infection **	28% (272)	23% (38)	28% (72)	17% (55)	**25% (437)**
Renal **	8% (75)	5% (8)	7% (17)	2% (5)	**6% (105)**

** p < 0.05

ETOH= alcohol, BAC = blood alcohol concentration, WD = withdrawal, CNS = central nervous system, GI = gastrointestinal.

**Table 4.** Complications associated with BAC in multivariate adjusted analysis.

	**OR**	**95% CI**	**p-value**
**Alcohol withdrawal**			
BAC < 100	12.02	7.0–20.7	<0.0001
BAC 100–199	16.81	10.4–27.2	<0.0001
BAC > 200	30.96	19.5–49.2	<0.0001
**Infectious**			
BAC < 100	0.73	0.44–1.20	0.2108
BAC 100–199	1.07	0.74–1.56	0.7104
BAC > 200	0.52	0.35–0.78	0.0013
**Coagulopathy**			
BAC < 100	0.81	0.46–1.42	0.4560
BAC 100–199	0.75	0.48–1.19	0.2241
BAC > 200	0.33	0.19–0.56	<0.0001
**Renal**			
BAC < 100	0.23	0.05–0.95	0.0419
BAC 100–199	1.01	0.54–1.90	0.9654
BAC > 200	0.20	0.07–0.57	0.0024
**CNS**			
BAC < 100	0.51	0.24–1.06	0.0717
BAC 100–199	0.28	0.14–0.56	0.0003
BAC > 200	0.41	0.23–0.71	0.0017

OR = odds ratio with reference to NO ETOH; CI = confidence interval; Adjustment variables: age, sex, use of safety device, position in vehicle, whether transferred or from the scene.
